# Book Review: Critical Thinking: A Concise Guide

**DOI:** 10.3389/fpsyg.2021.656331

**Published:** 2021-04-09

**Authors:** Valentin Gravet, Mathieu Hainselin

**Affiliations:** ^1^Département de Psychologie, Université de Picardie Jules Verne, Amiens, France; ^2^CRP-CPO, UR UPJV 7273, Université de Picardie Jules Verne, Amiens, France

**Keywords:** cognitive bias, belief, judgment, knowledge, open-mind, self-questioning

*“To believe or not to believe, that is the question”* should be an automatic question we ask ourselves. Thus, scientists' aim should be to provide reasons and evidence when many people do not believe in science. These kinds of questions are even more important during health crisis when the general population have to follow scientists' recommendations [i.e., coronavirus disease 2019 (COVID-19)]. Indeed, multiple factors can lead people to relay misinformation or be victim of false reasoning (Apuke and Omar, 2020). Bowell, Cowan, and Kemp's book (Bowell et al., 2020) is a great start to learn how to distinguish good arguments from false reasoning or rhetorical techniques. Synthesis and simplification of information, logical and analytical reasoning, as well as systematical evaluation of verbal content will be taught in this book, which come close to the very definition of critical thinking (Jacobs et al., 1997). To help the reader through the book, the authors made a chapter summary in the introduction and at the beginning and the end of each chapter. While some of the eight chapters are quite independent, a few of them are bonded together (3 and 4, 5, and 6).

## Evaluation of the Book's Content

The first chapter introduces us to the critical thinking with lots of definitions. Basics of argumentation, are explained and many practical examples (i.e., Martin Luther King's “I have a dream” speech) are put forward. Open-mindedness and self-questioning are explicitly promoted and encouraged.

Chapter 2 leads to a non-exhaustive list of rhetoric methods seeking to persuade without using arguments. Many tips are provided to spot these attempts in a speech and to judge the relevance of arguments without being under the influence of rhetorical elements. Overall, it is an easy-to-read chapter that teaches how to dodge non-argumentative ploys.

Both Chapters 3 and 4 are dedicated to logical reasoning. They are the most elaborated chapters of the book and introduce a lot of principles, models, and definitions. Chapter 3 starts with the question of deductive validity, which will be discussed through the concepts of true, false, valid, or invalid concerning arguments and their components. Chapter 4 introduces probabilistic reasoning and logic. Probabilities, mathematics models, and methods to judge the relevance of an argument are at the center of this chapter.

Again, both Chapters 5 and 6 are paired, as they are, respectively, dedicated to argument reconstruction and judgment. Longer than the other ones, Chapter 5 focuses on the process of extracting an argument in order to reconstruct it in its simplest form. Chapter 6 deals with argument analysis in two parts. The first part is about methods to assess both validity and relevance of a given argument. The second part includes some practical tips and advices to provide constructive criticism of an argumentation. After reading Chapter 6, you will be able to successfully pass the Ennis–Weir Critical Thinking Test (Ennis and Weir, 1985), a critical thinking test based on a flawed arguments letter.

The last two chapters are mostly independent from the rest of the book and are easy to read, although you do not have mathematical skills. Chapter 7 is probably the most on time chapter these days. It introduces pseudo-reasoning, fallacious, and misleading arguments (i.e., uses of *ad hominem* fallacy when responding to someone's argument by making an attack upon the person rather than addressing the argument itself). Beyond the concept, the authors explain a very interesting paradox: why these arguments should not be considered as reliable and why so many of us still tend to accept them.

The last chapter is a philosophical opening on epistemological and sociological questions. Concepts of truth or false, knowledge, and believing are discussed, leaving the reader to make up his own mind on the subject. The main purpose of this chapter is to add nuance to what we may consider as true, or not, even before analyzing logical structures and relevance of arguments.

## Discussion

Researchers in philosophy, psychology, and education agree that critical thinking covers skills of analysis, logical reasoning, judgment, and decision making (Lai et al., 2011). All these topics are explored in this book, allowing the reader to have an insight on what can be defined as critical thinking such as the mastery of language, logic, argumentation, and problem solving. Technical concepts are explained by different methods such as the schematization of arguments into syllogisms with premise(s) and conclusion(s) and the use of extended examples to decompose and analyze a speech. In addition, this fifth edition introduces the use of Venn diagrams to illustrate categorical deductive logic. Many detailed examples have also been added, as well as the discussion of current phenomena (i.e., fake news). We strongly encourage librarians and teachers to recommend this book to train critical thinking psychology students in university (Lacot et al., 2016) and earlier at school when possible (Hand et al., 2018). Indeed, from both practical and academic point of view, this book could be addressed to undergraduate students to enable them to develop an open-mindedness and a deep reflection around their own knowledge and the concepts addressed during their training and practice (i.e., therapies, models). Anyone, regardless of their previous knowledge, could benefit from this book, as there are lots of example, practical exercises and definitions. Finally, this book's additional contribution compared to previous books is to provide a methodical, simple, and complete explanation of the fundamental concepts related to critical thinking in a practical, playful, and concrete manner with numerous illustrations drawn from the real world. We hope this book will be translated in different languages in the future, as the flawed arguments and shortcuts are well-spread in the world.

## Author Contributions

VG wrote the manuscript. MH drafted it. All authors contributed to the article and approved the submitted version.

## Conflict of Interest

The authors declare that the research was conducted in the absence of any commercial or financial relationships that could be construed as a potential conflict of interest.
